# *Telenomus remus*, a Candidate Parasitoid for the Biological Control of *Spodoptera frugiperda* in Africa, is already Present on the Continent

**DOI:** 10.3390/insects10040092

**Published:** 2019-03-29

**Authors:** Marc Kenis, Hannalene du Plessis, Johnnie Van den Berg, Malick Niango Ba, Georg Goergen, Koffi Eric Kwadjo, Ibrahim Baoua, Tadele Tefera, Alan Buddie, Giovanni Cafà, Lisa Offord, Ivan Rwomushana, Andrew Polaszek

**Affiliations:** 1 CABI, 1 Rue des Grillons, 2800 Delémont, Switzerland; 2 IPM program, Unit for Environmental Sciences and Management, North-West University, 2520 Potchefstroom, South Africa; Hannalene.DuPlessis@nwu.ac.za (H.d.P.); Johnnie.VanDenBerg@nwu.ac.za (J.V.d.B.); 3 ICRISAT, BP 12404 Niamey, Niger; B.Malick@cgiar.org; 4 International Institute of Tropical Agriculture (IITA), 08 BP 0932 Tri Postal, Cotonou, Benin; g.goergen@cgiar.org; 5 University Nangui Abrogoua, 01 BP 3594 Abidjan 01, Côte d’Ivoire; kwadjoeric.sn@univ-na.ci; 6 University Dan Dicko Dankoulodo de Maradi, BP 465 Maradi, Niger; baoua.ibrahim@gmail.com; 7 International Center of Insect Physiology & Ecology (icipe), P.O. Box 30772-00100 Addis Ababa, Ethiopia; ttefera@icipe.org; 8 CABI, Bakeham Lane, Egham TW20 9TY, UK; a.buddie@cabi.org (A.B.); g.cafa@cabi.org (G.C.); l.offord@cabi.org (L.O.); 9 CABI Limuru Road, Muthaiga, PO Box 633-00621 Nairobi, Kenya; I.Rwomushana@cabi.org; 10 Natural History Museum, London SW75BD, UK; A.Polaszek@nhm.ac.uk

**Keywords:** biological control, egg parasitism, fall armyworm, invasive species, maize, *Spodoptera frugiperda*, *Telenomus remus*

## Abstract

The fall armyworm, *Spodoptera frugiperda*, a moth originating from tropical and subtropical America, has recently become a serious pest of cereals in sub-Saharan Africa. Biological control offers an economically and environmentally safer alternative to synthetic insecticides that are being used for the management of this pest. Consequently, various biological control options are being considered, including the introduction of *Telenomus remus*, the main egg parasitoid of *S. frugiperda* in the Americas, where it is already used in augmentative biological control programmes. During surveys in South, West, and East Africa, parasitized egg masses of *S. frugiperda* were collected, and the emerged parasitoids were identified through morphological observations and molecular analyses as *T. remus*. The presence of *T. remus* in Africa in at least five countries provides a great opportunity to develop augmentative biological control methods and register the parasitoid against *S. frugiperda*. Surveys should be carried out throughout Africa to assess the present distribution of *T. remus* on the continent, and the parasitoid could be re-distributed in the regions where it is absent, following national and international regulations. Classical biological control should focus on the importation of larval parasitoids from the Americas.

## 1. Introduction

The fall armyworm, *Spodoptera frugiperda* (JE Smith) (Lepidoptera: Noctuidae) is a highly destructive pest of cereals, and is a native of the tropical and sub-tropical regions of North, Central, and South America [1]. First detected on the African continent in January 2016 in Nigeria [2], *S. frugiperda* has now been reported in almost all of sub-Saharan Africa [3]. Recently published pest distribution and climatic suitability models have indicated that the environmental requirements for this pest to establish itself permanently are present through large parts of Africa and Asia and some parts of Europe [4,5]. The pest’s distribution has reached the southern fringes of the Sahara, and it has most recently been reported from several states in India [6,7], as well as Yemen, Myanmar, Thailand, and Sri Lanka [8]. Indeed, it is likely that it will spread further north to Europe and other countries in Asia. The invasion of *S. frugiperda* threatens the food security of more than 200 million people in Africa whose main staple crop is maize. Based on preliminary estimates in 12 African maize-producing countries, in the absence of proper control methods, *S. frugiperda* has the potential to cause maize yield losses of 8.3 to 20.6 million tonnes per annum. The value of these losses is estimated at between US$ 2.5 to 6.2 billion, with over US$ 13 billion worth of crops at risk [9]. In addition, the pest, known to be highly polyphagous, is likely to jeopardize the trade and export of other crops from the invaded regions. 

Following the invasion of *S. frugiperda* into Africa, emergency responses have been geared towards the use of chemical insecticides [10]. The frequent application of different classes of insecticides is unsustainable in the long-run because it leads to the development of insecticide resistance, increases production costs, and causes biodiversity and environmental impacts as well as health risks to the growers and consumers [11,12]. It also disrupts IPM measures, such as biological control, targeted at other cereal pests [9,13]. Therefore, it is important to minimize the use of insecticides, especially the highly hazardous and broad-spectrum ones, and to develop, promote, and deploy proven and sustainable IPM technologies against *S. frugiperda*. 

Biological control, i.e., the use of natural enemies to control a pest, is central to the development of IPM systems. Three different biological control strategies can be envisaged against *S. frugiperda* in Africa [14]. Firstly, natural enemies could be imported from the native area of the pest for release and permanent establishment in Africa (classical biological control). Secondly, natural enemies could be mass produced for regular releases and temporary control (augmentative biological control). Thirdly, the action of natural enemies already present in the crops could be enhanced by the application of various cultural practices including the use of selective insecticides (conservation biological control).

Parasitoids are natural enemies most commonly used against insect pests [15]. Over 150 parasitoid species have been reported to attack *S. frugiperda* in its native range in the Americas [13,16,17]. Among these, *Telenomus remus* Nixon (Hymenoptera: Platygastridae) is an egg parasitoid of various Lepidoptera species, originating from peninsular Malaysia (but see the discussion section, under “Taxonomy of *Telenomus remus*”). It was introduced against *Spodoptera* spp. to various parts of the world, including India, Pakistan, Australia, New Zealand, the Caribbean, Colombia and Venezuela. The parasitoid is now found in most of the distribution range of *S. frugiperda* in the Americas [18]. In Africa, it was also released in the Cape Verde Islands in the early 1980s, but its establishment has not been confirmed [19]. In the Americas, parasitism due to natural populations of *T. remus* is moderate but it is used successfully as an augmentative biological control agent in several countries [20,21,22]. *T. remus* can be produced under laboratory conditions on *S. frugiperda* or other hosts and released in the field [10,20,23]. A female produces an average of 270 eggs during her lifespan, usually laid individually in each host egg, avoiding superparasitism [20]. They are also able to parasitize the whole egg mass, whereas other egg parasitoids such as *Trichogramma* spp. tend to parasitize only the external layer [24]. Augmentative releases of *T. remus* in maize fields can result in 80–100% parasitism, providing full control of *S. frugiperda* [20,21,22,25]. The main challenge for a wider utilisation of *T. remus* is the difficulty to mass produce its natural hosts, and the need for developing rearing systems on factitious hosts [23,26]. Nevertheless, *T. remus* is considered for introduction into Africa as part of the response to *S. frugiperda* [10].

In this paper, we report on the observation that *T. remus* is already present in Africa, speculate how it might have arrived on the continent, and assess the potential for its use in augmentative biological control in Africa. This is the first report of an egg parasitoid of *S. frugiperda* in the open field in Africa.

## 2. Materials and Methods 

### 2.1. Field Sampling

Parasitized eggs of *S. frugiperda* were obtained from eight sites in five countries: Benin (2 sites), Côte d’Ivoire (1), Kenya (1), Niger (2), and South Africa (2), in 2017 and 2018 (Table 1). These collections were made as part of different, unrelated projects, during *S. frugiperda* population surveys or for sampling egg masses for laboratory rearing. Quantitative data on parasitism were not collected, as it was not the primary objective of these surveys. Sampling and rearing techniques varied between sampling sites, but in all cases egg masses of *S. frugiperda* were brought to the laboratory and held in small containers. Egg parasitoids that emerged from egg masses were removed from the containers and killed by immersion in 70–96% alcohol. Larvae from the non-parasitized eggs in the same egg mass were reared until adult emergence to confirm the identity of the hosts.

Samples of the egg parasitoids were sent to the Centre for Agriculture and Bioscience International (CABI) and the Natural History Museum, London, UK (NHMUK) for morphological and molecular analyses. Voucher specimens of egg parasitoids from all localities are deposited permanently in NHMUK, the North-West University, Potchefstroom, South Africa; and CABI, Delémont, Switzerland.

### 2.2. Morphological Analysis

Identifying *Telenomus* species is particularly difficult among other minute parasitoids. Females tend to appear very similar between species, with only very few useful morphological characters. Male genitalia, however, provide fairly reliable characters for species identification. Specimen preparation and dissection and mounting of genitalia are relatively straightforward [27], and can be done successfully on specimens from which DNA has already been extracted [28]. Several male specimens (> 3) from each locality were dissected and examined.

### 2.3. Molecular Analyses

Thirteen specimens, obtained from all but one site (Maradi, Niger), were analysed (Table 1). To obtain barcodes (around 600 bp of the mitochondrial gene (mtDNA) cytochrome oxidase c subunit 1 (COI)) from the samples, we followed the protocols described in [29]. These included the isolation of DNA from fragments of the abdomen of each specimen, by adding 20 µL of microLYSIS^®^-PLUS (MLP; Microzone Ltd., Haywards Heath, UK) to the material. The suspension was macerated with a sterile micropestle (VWR International Ltd., UK) to facilitate the disruption of the exoskeleton and tissues of the samples. DNA was then liberated into the MLP by placing the sample tubes in a thermal cycler and subjecting them to the heat profile recommended by the manufacturer for difficult samples. PCR reactions were carried out using a Hybaid PCR Express thermal cycler in heated-lid mode. Amplifications were carried out in 0.5 ml microcentrifuge tubes in 20 µL reactions containing: 1 µL MLP DNA extract; primers LCO1490 and HCO2198 (5’-GGTCAACAAATCATAAAGATATTGG-3’ and 5’-TAAACTTCAGGGTGACCAAAAAATCA-3’, respectively; [30]), each at 150 nM; and 10 μL of MegaMix-Royal (Microzone Ltd, Haywards Heath, UK) mastermix solution, containing optimized mixture of *Taq* polymerase in 2 × enhancing buffer (6 mM MgCl_2_), with 400 μM dNTPs and blue MiZN loading dye. Reactions were made up to a final volume of 20 μL with sterile molecular grade H_2_O. PCR reactions were preincubated for 5 min at 95 °C followed by 39 cycles of 30s at 94 °C, 30s at 51 °C, and 75s at 72 °C. Samples were finally incubated for 10 min at 72 °C followed by chilling at 10 °C. A second round of amplification (i.e., “reamplification”) was undertaken as follows: 1 µL of each of the above PCR products was used as a template. The reaction was carried out under the same conditions, with the exception of the number of cycles, which were reduced to 30. PCR products were visualized with gel electrophoresis, purified by microCLEAN (Microzone Ltd., Haywards Heath, UK), and resuspended in 15µL sterile molecular grade H_2_O. After sequencing reactions, excess unincorporated dye terminators were removed by means of DyeEx 2.0 (Qiagen, UK) gel filtration columns, according to the manufacturer’s instructions. Eluted samples were resuspended in 16 µL highly deionized formamide (HiDi™; ThermoFisher Scientific, Gloucester, UK). Sanger sequencing was carried out on an ABI 3130 Genetic Analyzer (ThermoFisher Scientific, Gloucester, UK) in accordance with the manufacturer’s instructions.

Sequences were aligned using the multiple sequence alignment plug-in CLUSTALW in MEGA7 [31]. Sequences obtained in this study were compared with authenticated sequences available from the Barcoding of Life Data system [32] and additional sequences from the GenBank^®^ data base [33] and trimmed to size. The evolutionary history was inferred by using the maximum likelihood method based on the Tamura–Nei model [34]. Initial tree(s) for the heuristic search were obtained automatically by applying Neighbor–Join and BioNJ algorithms to a matrix of pairwise distances estimated using the maximum composite likelihood (MCL) approach, and then selecting the topology with superior log likelihood value. Codon positions included were 1st+2nd+3rd+noncoding. All positions containing gaps and missing data were eliminated. Trees were drawn to scale, with branch lengths measured in the number of substitutions per site. The bootstrap consensus tree inferred from 1000 replicates is taken to represent the evolutionary history of the taxa analysed [35]. Branches corresponding to partitions reproduced in less than 50% bootstrap replicates are collapsed. The percentage of replicate trees in which the associated taxa clustered together in the bootstrap test (1000 replicates) are shown next to the branches [35]. Evolutionary analyses were conducted in MEGA7 [31].

## 3. Results

Morphological analysis of several male specimens from each of the eight collection localities suggested identical species with almost no variation. Specimens were compared with a series of *T. remus* from many localities in Asia and the Americas, and found to be conspecific. A single specimen of *T. remus* in the NHMUK collection from Kenya, reared from eggs of *Spodoptera triturata* (Walker), was identified as *T. remus* by the last author in 1988 [36]. Records of *T. remus* from Serbia (Europe) in the Hymenoptera online database [37] are actually from Venezuela. The specimen data read as follows: “*Ex. huevo de Spodoptera spp.; Cria masiva Lab. Serbio; (Barquisimeto, Venezuela-Lara-m.) (30.VIII.1990)”*. “Serbio” is clearly the source of the error.

**Figure 1:**
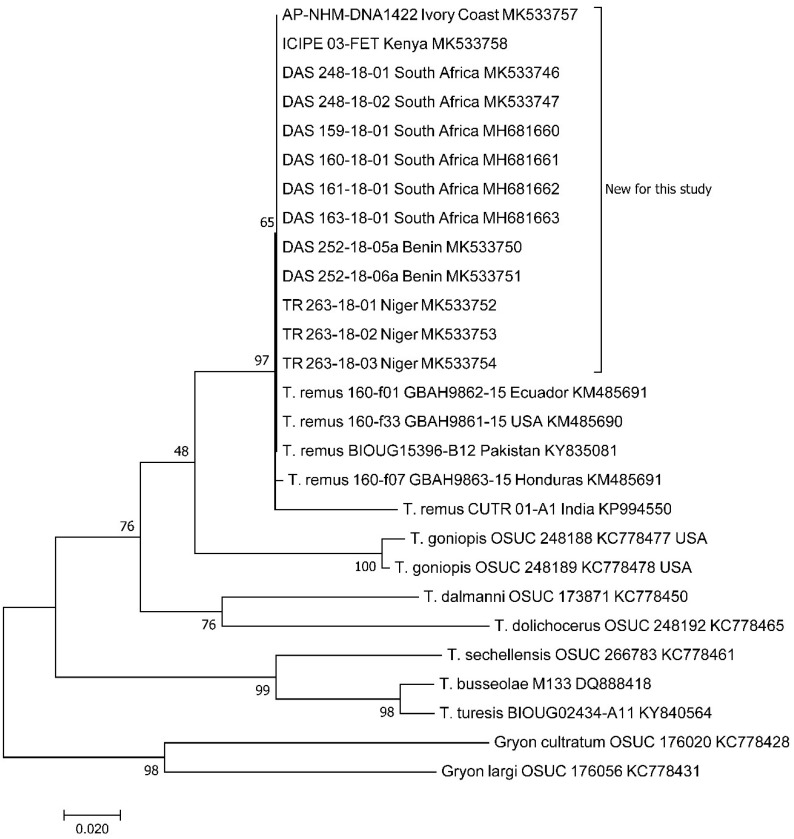
Molecular Phylogenetic analysis of *Telenomus remus* and related species by maximum likelihood method. The tree with the highest log likelihood (-1910.13) is shown. The percentage of trees in which the associated taxa clustered together is shown next to the branches. The analysis involved 27 nucleotide sequences. There were a total of 382 positions in the final dataset. The 13 specimens analysed in this study are indicated as “New for this study”, and their codes are indicated in Table 1.

The thirteen barcode sequences derived from this study gave 100% match to each other and were identified by comparison with those available from the BOLD database. The sequences were deposited in GenBank with accession numbers MH681660-3 and MK533746-7; MK533750-4; MK533757-8. Over 99% of the pairs of bases were identical to a series of 31 specimens in BOLD identified as *Telenomus remus*. Matches varied from 99.05% to 99.63%, with 27 specimens from Bangladesh, and were 99.62%, 99.56%, 99.56%, and 99.34% with single specimens from Pakistan, Ecuador, USA (Florida), and Honduras, respectively, but only 96.3% with a *T. remus* specimen from India (Kerala). The samples from Honduras, Ecuador, and Florida are those described in detail in Hay-Roe et al. [38]. The most closely-related *Telenomus* species, for which barcodes are publicly available, is *Telenomus goniopis*, which overlaps with our specimens at 91.17–91.33% (Figure 1). There is a strong probability that the “*T. remus*” sequence from India is based on a misidentification.

## 4. Discussion

### 4.1. Presence of T. remus in Africa

This study shows that a species identified from both morphology and DNA sequences as *T. remus* has been attacking *S. frugiperda* eggs in at least five countries in West, East, and Southern Africa, and eggs of the lawn worm, *S. triturata,* in Kenya. The genetic distance between the specimens from Africa and the numerous Asian and American *T. remus* specimens available in the barcode datasets is sufficiently small (< 1%) to conclude that it is the same species as the one that was released previously in South Asia and the Americas. Even though differences between intra- and interspecific divergence strongly vary between taxonomic groups, and genetic distances have to be used with caution to define species, Virgilio et al. [39] observed that 95% of mean interspecific congeneric distances in insects were found in the interval 2.47–21.00%. In an unpublished thesis, Bowers [40] suggested that, in *Telenomus* spp., intra-specific barcode divergences were below 1.6% whereas interspecific divergences were greater than 5.6%.

It is not known when and how *T. remus* arrived in Africa, but it certainly arrived before *S. frugiperda* since a specimen was collected in Kenya in 1988. This species parasitizes several *Spodoptera* spp. and some other Noctuidae [41], and could have remained unnoticed on other hosts for a long time because the adults of *Telenomus* spp. are extremely small and difficult to distinguish and identify on a morphological basis [38].

### 4.2. Implications for the Biological Control of S. frugiperda

No matter the pathway of introduction, the presence of *T. remus* has important implications for the biological control of *S. frugiperda* in Africa. Firstly, it shows that the parasitoid complex of *S. frugiperda* in Africa should be studied in detail before the introduction of exotic parasitoids. Prior to this report, the only published record of field parasitism of *S. frugiperda* in Africa was by Sisay et al. [42] in East Africa, but they did not report *T. remus* or any other egg parasitoid, with the exception of the egg-larval parasitoid *Chelonus curvimaculatus* Cameron, found at two locations. There are efforts underway for importing parasitoids from the native range of *S. frugiperda* for releases in Africa, either for permanent establishment and control or for augmentative biological control. *Telenomus remus* is high on the list of priority species to introduce, because it is the main egg parasitoid of *S. frugiperda* in its native range and, especially, because it is already successfully used in augmentative biological control programs and therefore can easily be adapted to the African situation. Our finding that *T. remus* is already extant in Africa suggests that importation efforts should focus on larval or pupal parasitoids, since the other known egg parasitoids of *S. frugiperda* in America (mostly *Trichogramma* spp.) are too polyphagous to be considered for introduction into Africa and not as effective. 

A specific research programme should be developed for *T. remus* in Africa. Surveys for egg parasitoids should be carried out to determine how widespread *T. remus* is in Africa, and parasitism rates should be properly assessed to evaluate its impact on *S. frugiperda* populations. A phylogeographic study using molecular tools, such as in [43], would help in clarifying the history of *T. remus* in Africa. These surveys should also determine which other African Lepidoptera species served as host for *T. remus* prior to the arrival of *S. frugiperda*. For instance, the single record of *T. remus* on *S. triturata* needs to be followed up closely. Knowing its host range in African agroecosystems may also support the development of measures to enhance the parasitoid in a conservation biological control programme. Surveys for egg parasitism in Africa may also reveal that other *Telenomus* spp. have adopted *S. frugiperda,* in which case their interactions with *T. remus* should be investigated. In Africa, at least 10 *Telenomus* species have been found on cereal stem borers and have been well studied [27,44]. In contrast, those attacking *Spodoptera* spp. are largely unknown.

If the distribution range of *T. remus* in Africa does not fully overlap that of *S. frugiperda*, the egg parasitoid could be redistributed in the regions where it is absent, following national and international regulations for introduction of natural enemies. Climate modelling, similar to [4] for *S. frugiperda*, should determine whether all areas in Africa are suitable for its establishment. Several studies have assessed its climatic requirements, e.g., [26,45,46,47], which should allow for the development of such models. Methods to use *T. remus* in augmentative biological control programmes should be developed based upon those used in the Americas [10,20,23,25,26], and *T. remus* should be registered as biological control agent against *S. frugiperda*. However, local specificities need to be considered, e.g., the fact that, in the Americas, maize is often planted in large monocultures while most of the maize in Africa is cultivated by smallholder farmers in mixed systems. Challenges may also be encountered in the mass production of the parasitoid due to the inherent difficulty of rearing *T. remus* on its natural host and the need of a factitious host for mass production [23,26,48]. Since the *T. remus* populations established in Africa may have never encountered *S. frugiperda* until recently, and because it might have become adapted to completing development in yet unknown hosts, it may be less efficient on this host than populations from the Americas that have evolved with *S. frugiperda* during, at least, the past 40 years. Therefore, it would be interesting to compare *T. remus* populations of African and American origins regarding their performance on *S. frugiperda* in quarantine. If American populations appear significantly more efficient than the African populations, the introduction of American populations could be considered.

### 4.3. Taxonomy of Telenomus remus

*Telenomus remus* was described from Ulu Gombak, just NE of Kuala Lumpur, Malyasia [49]. In the original description, Nixon states: ‘’This species is probably *Telenomus spodopterae* Dodd, which was described from four females labelled “from eggs of a moth *Spodoptera* sp. on sugar beet, Krebet, Java, 23.7.1913”. Nixon was reluctant to use Dodd’s name, as he considered the original description inadequate and thought there might be an important difference in the fore wing proportions. At least two more *Telenomus* species appear morphologically indistinguishable from *T. remus* (including male genitalia): *Telenomus nawai* Ashmead was described from Gifu, Japan [50]; and *T. soudanensis* (Risbec) was described from West Africa (probably Mali) [51]. There is some likelihood that all of these named species are conspecific [44]. Further research to establish the identities and possible conspecificity of the three described species would focus on DNA sequencing of specimens collected recently from the three type localities. Two independent studies have found mating incompatibility between two apparent species: Raveendranath [52] found a population from Hawaii was reproductively incompatible with one from Barbados, and Arakaki et al. [53] found an isolated population of *T. nawai* in Japan that is thelytokous due to *Wolbachia* infection. Further research is needed to clarify the status of these species.

## 5. Conclusions

This study showed that *T. remus,* the main egg parasitoid of *Spodoptera frugiperda* in the Americas that is considered for introduction into Africa, has now been found in East, South, and West Africa. This finding has important implications for the development of augmentative biological control and IPM programmes against *S. frugiperda* in Africa.

## Figures and Tables

**Table 1:** Details of the sampling sites, numbers, and accession numbers of specimens sequenced.

Country	Locality (Province)	Coordinates	Collection Date	Host Plant	No. Barcoded	Code (as in Figure 1)
South Africa	Mbombela (Mpumalanga)	25.442149°S 30.992122°E	20.04.18	Maize	4	DAS 160-18-01 DAS 161-18-01 DAS 163-18-01 DAS-248-18-01
	Malelane (Mpumalanga)	25.595231°S 31.665183°E	20.04.18	Maize	2	DAS-159-18-01 248-248-18-02
Côte d’Ivoire	Yamoussoukro (Kami)	6.875833°N 5.363333°W	25.05.18	Maize	1	AP-NHM-DNA1422
Niger	Maradi (Djiratawa)	13.2360°N 7.0760°E	15.08.17	Maize	0	1455 (NHM)
	Tilabéri (Sadoré)	13.2454°N 2.3047°E	18.09.18	Sorghum	3	TR 263-18-01 TR 263-18-02 TR 263-18-03
Benin	Abomey Calavi (Atlantique)	6.4375°N 2.3283°E	04.07.18	Maize	1	DAS 252-18-05a
	Abomey Calavi (Atlantique)	6.43064°N 2.29544°E	01.10.18	Maize	1	DAS 252-18-6a
Kenya	Kilifi (Kilifi)	3.51.06°S 39.9092°E	01.10.18	Maize	1	03-FET
